# Acute sublethal exposure to toxic heavy metals alters honey bee (*Apis mellifera*) feeding behavior

**DOI:** 10.1038/s41598-019-40396-x

**Published:** 2019-03-12

**Authors:** Christina M. Burden, Mira O. Morgan, Kristen R. Hladun, Gro V. Amdam, John J. Trumble, Brian H. Smith

**Affiliations:** 1 Arizona State University, School of Life Sciences, Tempe, Arizona USA; 2 Present Address: Union College, Division of Science and Mathematics, Lincoln, Nebraska 68505 USA; 3 University of California – Riverside, Department of Entomology, Riverside, California USA; 4 Norwegian University of Life Sciences, Faculty of Environmental Sciences and Natural Resource Management, Aas, Norway

## Abstract

Heavy metal toxicity is an ecological concern in regions affected by processes like mining, industry, and agriculture. At sufficiently high concentrations, heavy metals are lethal to honey bees, but little is known about how sublethal doses affect honey bees or whether they will consume contaminated food. We investigated whether honey bees reject sucrose solutions contaminated with three heavy metals – cadmium, copper, and lead – as a measure of their ability to detect the metals, and whether ingesting these metals altered the bees’ sucrose sensitivity. The metals elicited three different response profiles in honey bees. Cadmium was not rejected in any of the assays, and ingesting cadmium did not alter sucrose sensitivity. Copper was rejected following antennal stimulation, but was readily consumed following proboscis stimulation. Ingestion of copper did not alter sucrose sensitivity. Lead appeared to be palatable at some concentrations and altered the bees’ sensitivity to and/or valuation of sucrose following antennal stimulation or ingestion of the metal. These differences likely represent unique mechanisms for detecting each metal and the pathology of toxicity. The bees’ ability to detect and consume these toxic metals highlights the risk of exposure to these elements for bees living in or near contaminated environments.

## Introduction

In multiple regions around the world, the soil and water reservoirs are contaminated with heavy metals, especially within and surrounding urbanized and industrialized areas, mining and fossil fuel extraction sites, and heavily-used agricultural regions^1–6^. Many of these heavy metals are taken up by the plants growing in contaminated soil and show elevated levels in plant tissues as compared to plants grown in control soils^7–12^. Plants such as crop radish (*Raphanus sativus*), common flax (*Linum usitatissimum*), hemp (*Cannabis sativa* L.), cotton (*Gossypium hirsutum* L.), and milkwort jewelflower (*Streptanthus polygaloides*) have been shown to accumulate heavy metals such as copper, cadmium, lead, zinc, and nickel in their leaves and flowers^7,9,12,13^. Furthermore, the accumulation of cadmium, copper, and lead in crop radish was shown to be sufficient to negatively affected plant survival and productivity^7,12^.

In addition to affecting plant productivity and survival, environmental contamination with heavy metals exposes pollinators that depend on these plants to potentially toxic levels of the metals. This can cause a reduction in species diversity, brood growth, and survival of wild and managed pollinator species, as has been shown in areas known to have elevated levels of metal contamination^14,15^. The presence of some metal contaminants – such as manganese, aluminum and nickel – in flowers has also been shown to alter the frequency of visits by pollinators and negatively impact their navigation abilities^9,15–18^. The effect on pollinator activity and survival may be substantial even when concentrations of heavy metals are lower than minimal risk levels for human health as stipulated by regulatory institutions such as the Agency for Toxic Substances and Disease Registry (https://www.atsdr.cdc.gov).

Understanding how environmental pollution with heavy metals affects one pollinator species, the European honey bee (*Apis mellifera*), is of special concern since they are important for the pollination of approximately 70% of food crops^19^. Previous studies have shown that honey, propolis, and wax in colonies around the world contain multiple toxicants – including insecticides, fungicides, herbicides, and some heavy metals^20,21^. However, the sensitivity and vulnerability of this pollinator species to many of these contaminants, or to combinations of the contaminants, is not well understood.

Honey bees may be able detect some toxicants through receptors on their antennae and proboscis. Bees have been shown to reject sucrose solutions contaminated with quinine and concentrated sodium chloride upon stimulation of the antennae or proboscis, presumably because of an unpalatable “taste”^22^. Some of these substances have been shown to activate receptors on the honey bee proboscis differentially from sucrose stimulation^22,23^. Therefore, these substances may be recognized as harmful through the way the honey bee perceives the “taste” of the substance.

Honey bees also may be able to recognize a substance as harmful through the induction of malaise following ingestion. The animals then may associate the sensory perception of the substance with the malaise and, through conditioned taste aversion, learn to avoid it in the future^24,25^. For example, honey bees fed with the toxin amygdalin soon learned to avoid solutions containing the toxin, and to reduce their feeding response to olfactory cues associated with exposure to the toxin^22^. However, not all toxicants may induce malaise at the concentrations honey bees are exposed to in the environment.

There are some toxicants that honey bees do not appear to be able to detect through their antennae or proboscis. For example, selenium, a metalloid that is toxic at high concentrations, does not appear to be detected through stimulation of receptors on the antennae or the proboscis^26^. Honey bees readily consume sucrose contaminated with even lethal concentrations of selenium^26^. Honey bees are particularly at risk to those toxicants they are unable to detect or unable to recognize as harmful.

Investigating the likelihood that honey bees will readily feed on metal contaminated resources helps determine the level of threat a metal poses to the honey bee population. If the honey bees are able to detect and reject the metal in their food and water sources through the negative sensory experience with the metal or learning to avoid it via conditioned taste aversion, that metal poses a somewhat lower risk to the foraging bees and their colony.

Three heavy metals that have been detected at high levels in the environment are cadmium, copper, and lead. In major agricultural regions of the United States soil concentrations of these metals can range widely (cadmium: < 0.01–2 mg/kg, copper: < 0.06–495 mg/kg, lead: < 1.0–135 mg/kg)^27^. More information on the distribution of these metals in soils can be found through the United States Geographical Survey (https://mrdata.usgs.gov/geochem/doc/averages/countydata.htm). Common flax, hemp, and cotton all have been shown to bioaccumulate elevated levels of cadmium, copper, and lead in their leaves and flower parts when grown in soil containing concentrations of these metals similar to these published ranges^13,28^. Consequently, these metals would potentially be made available to pollinators foraging in regions with similar levels of contamination and plant species exhibiting a similar amount of bioaccumulation.

All of these metals also bioaccumulate in adult and larval honey bees and the colony’s honey, wax, and propolis supplies^29,30^. These metals all have significant negative effects on individual honey bee health and survival, as well as on the whole colony, when present at toxic levels^29,30^. However, it is still not known if bees are able to detect or reject toxic levels of these metals in a food source. Consequently, at this time it is difficult to assess the exposure risk of these metals for honey bees colonies near contaminated areas.

Hladun, *et al*.^26^ used a series of assays to determine whether honey bees can detect the presence of a metalloid – selenium – at toxic levels in a food source and whether the presence of or the previous consumption of the contaminant is sufficient to alter their motivation to feed. These assays are based on the proboscis extension reflex (PER), a reflexive extension of the feeding apparatus that is exhibited by honey bees when their antennae are stimulated with a sufficiently motivating food source. We used these same assays to determine if honey bees are able to detect and reject heavy metal contaminants in a food source.

We tested honey bees’ likelihood of rejecting toxic levels of heavy metals in sucrose to assess the degree of risk environmental contamination with these metals poses to honey bee health and survival. We used antennal and proboscis stimulation with the contaminated sucrose to determine if they are able to reject contaminated food prior to ingestion. We also investigated the possibility of post-ingestional rejection of the contaminated food based on the potential induction of malaise from ingestion of a toxic quantity of the metals.

## Results

In each of the assays reported, we measured the honey bees’ ability to detect and reject sucrose contaminated with heavy metals. For antennal stimulation assays, we used the presence or absence of PER following stimulation to assay rejection of the contaminated sucrose. To assess the ability to detect the contaminant via proboscis stimulation, we measured consumption of the contaminated sucrose. To determine if consumption of the contaminated sucrose induced malaise, we used the presence or absence of PER in response to antennal stimulation with uncontaminated sucrose. This test was performed 2–3 hours following ingestion of the metal.

### Rejection of metal contaminated sucrose following antennal and proboscis stimulation

**Figure 1:**
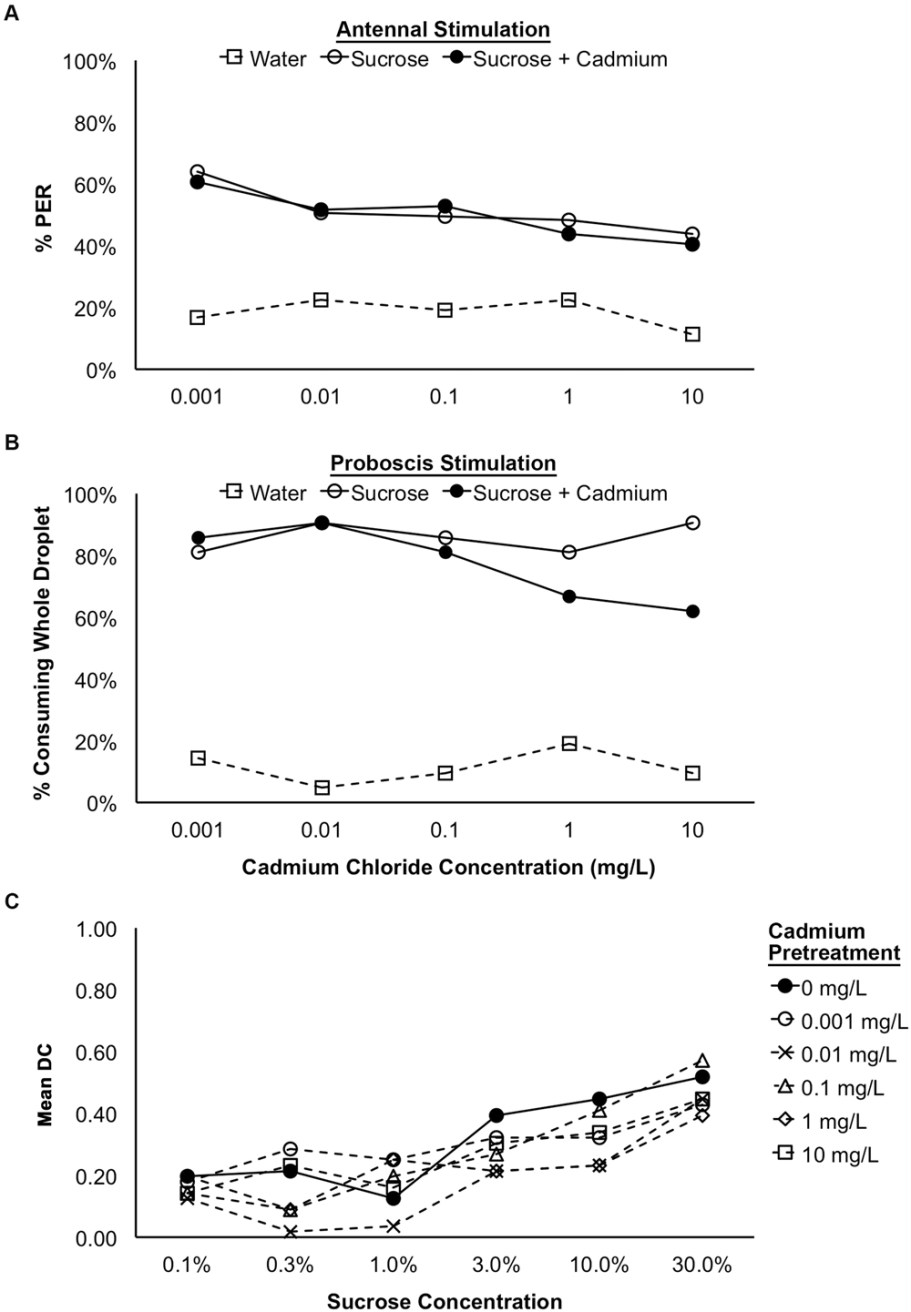
The responsiveness of worker honey bees to antennal (**A**; n = 89) and proboscis (**B**; n = 21/treatment group) stimulation with water, sucrose, and sucrose contaminated with cadmium chloride and the effect of pretreatment with cadmium chloride on the sucrose response threshold in treated and control bees (**C**; n = 28/treatment group). For the assay testing the bees’ responsiveness to antennal stimulation with cadmium-contaminated sucrose solutions and the assay testing the sucrose response threshold following cadmium pretreatment, the percentages of bees exhibiting the proboscis extension reflex (% PER) are shown. For the assay testing the bees’ responsiveness to proboscis stimulation with cadmium-contaminated sucrose solutions, the percentages bees that consumed the whole droplet of each test solution (% consuming whole droplet) are shown. For the assay testing sucrose response thresholds in pretreated and control bees, mean DC indicates the mean value of the discrimination code. Bonferroni-corrected pairwise comparisons are indicated: ***p < 0.001, **p < 0.01, *p < 0.05.

Honey bees did not exhibit significant rejection of sucrose solutions contaminated with cadmium chloride following antennal or proboscis stimulation. During the antennal stimulation assay, there was no difference in the percentage of bees responding to the sucrose and the sucrose + cadmium solutions for any of the concentrations of cadmium tested (Fig. 1A; Logistic GEE; SOLUTION: χ^2^ = 0.384, p = 0.535; CONCENTRATION: χ^2^ = 15.975, p = 0.003). Similarly, during the proboscis stimulation assay, there was no effect of cadmium concentration on the percentage of bees consuming the sucrose and sucrose + cadmium solutions (Fig. 1B; Logistic GEE: CONCENTRATION: χ^2^ = 2.033, p = 0.730; SOLUTION: χ^2^ = 2.315, p = 0.128). However, there was a non-significant reduction in the percentage of bees consuming the sucrose + cadmium solution at the two highest concentrations (1 mg/L and 10 mg/L; Fig. 1B).

**Figure 2:**
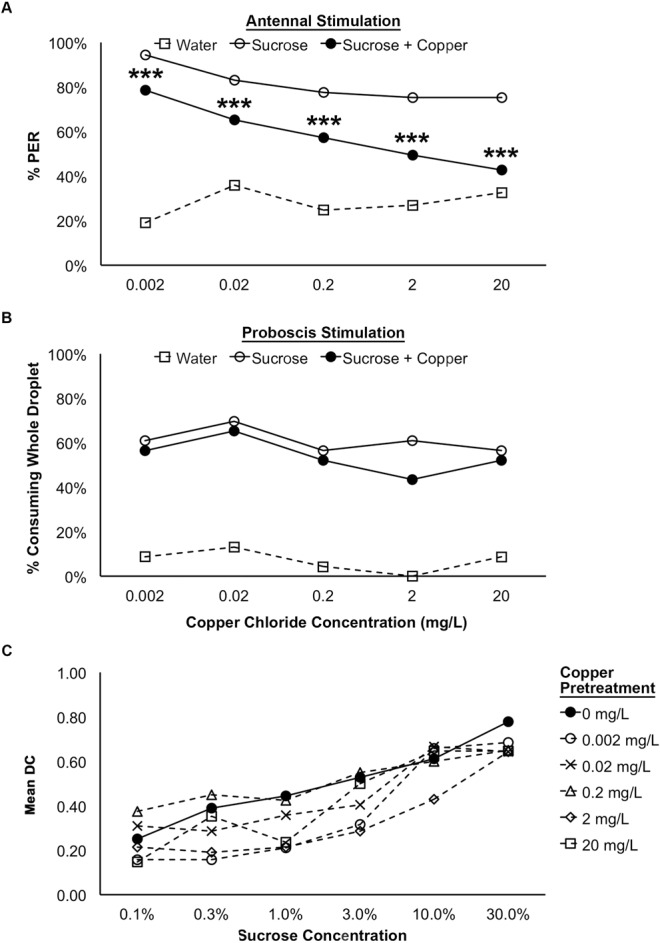
The responsiveness of worker honey bees to antennal (**A**; n = 89) and proboscis (**B**; n = 23/treatment group) stimulation with water, sucrose, and sucrose contaminated with copper chloride and the effect of pretreatment with copper chloride on the sucrose response threshold in treated and control bees (**C**; 0 mg/L: n = 18, 0.002 mg/L: n = 19, 0.02 mg/L: n = 21, 0.2 mg/L: n = 20, 2 mg/L: n = 21, 20 mg/L: n = 17). For the assay testing the bees’ responsiveness to antennal stimulation with copper-contaminated sucrose solutions and the assay testing the sucrose response threshold following copper pretreatment, the percentages of bees exhibiting the proboscis extension reflex (% PER) are shown. For the assay testing the bees’ responsiveness to proboscis stimulation with copper-contaminated sucrose solutions, the percentages bees that consumed the whole droplet of each test solution (% consuming whole droplet) are shown. For the assay testing sucrose response thresholds in pretreated and control bees, mean DC indicates the mean value of the discrimination code. Bonferroni-corrected pairwise comparisons are indicated: ***p < 0.001, **p < 0.01, *p < 0.05.

In contrast, the presence of copper chloride in the sucrose solution significantly affected the percentage of bees exhibiting PER during the antennal stimulation assay (Logistic GEE; CONCENTRATION: χ^2^ = 56.283, p < 0.001; SOLUTION: χ^2^ = 71.499, p < 0.001). There was a significant reduction in the percentage of bees exhibiting PER to all solutions containing copper as compared to the uncontaminated sucrose solution (Fig. 2A; Bonferonni-corrected pairwise comparisons; 0.002 mg/L: p < 0.001, 0.02 mg/L: p < 0.001, 0.2 mg/L: p < 0.001, 2 mg/L: p < 0.001, 20 mg/L: p < 0.001). The magnitude of this reduction increased with increasing concentration of copper in the solution (Fig. 2A). There was, however, no effect of copper on consumption of the contaminated sucrose solution for any of the concentrations of copper tested following proboscis stimulation (Fig. 2B; Logistic GEE; CONCENTRATION: χ^2^ = 2.003, p = 0.730; SOLUTION: χ^2^ = 2.315, p = 0.128).

**Figure 3:**
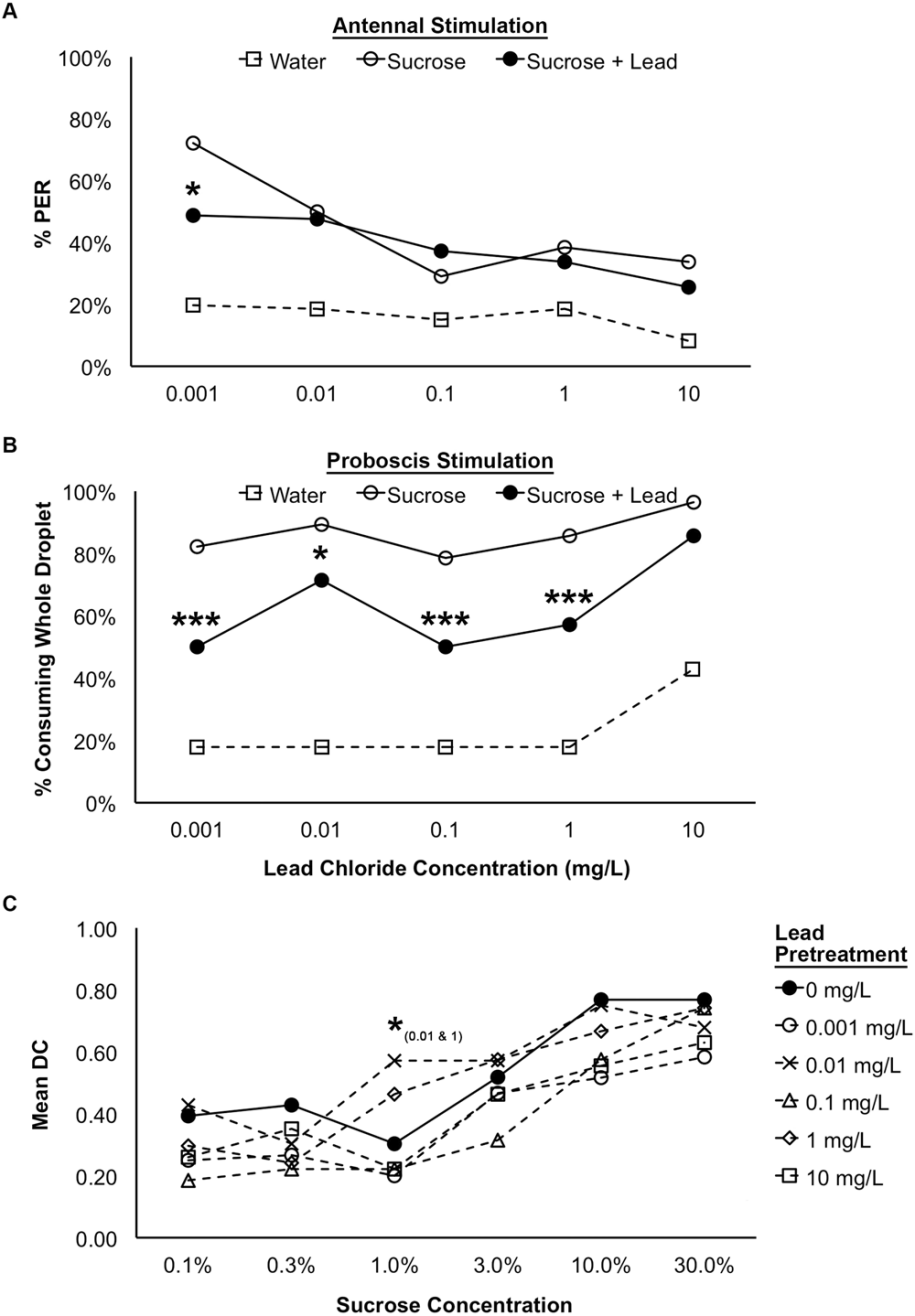
The responsiveness of worker honey bees to antennal (**A**; n = 86) and proboscis (**B**; n = 28/treatment group) stimulation with water, sucrose, and sucrose contaminated with lead chloride and the effect of pretreatment with lead chloride on the sucrose response threshold in treated and control bees (**C**; 0 mg/L: n = 28, 0.001 mg/L: n = 30, 0.01 mg/L: n = 28, 0.1 mg/L: n = 27, 1 mg/L: n = 27, 10 mg/L: n = 27). For the assay testing responsiveness to antennal stimulation with lead-contaminated sucrose solutions, and the assay testing the sucrose response threshold following lead pretreatment, the percentages bees exhibiting the proboscis extension reflex (% PER) are shown. For the assay testing the bees’ responsiveness to proboscis stimulation with lead-contaminated sucrose solutions, the percentages of bees that consumed the whole droplet of each test solution (% consuming whole droplet) are shown. For the assay testing sucrose response thresholds in pretreated and control bees, mean DC indicates the mean value of the discrimination code. Bonferroni-corrected pairwise comparisons are indicated: ***p < 0.001, **p < 0.01, *p < 0.05 for antennal and proboscis response assays. Significance levels for model contrasts are indicated (***p < 0.001, **p < 0.01, *p < 0.05) for sucrose response threshold analysis.

Lead chloride contaminated solutions elicited different patterns of responses than the other two metals. During the antennal stimulation assay, there was a significant effect of lead concentration on the percentage of bees exhibiting PER to sucrose only and sucrose + lead stimulation and a significant interaction between the solutions tested (sucrose only vs. sucrose + lead) and the concentration of lead in the sucrose + lead solution (Fig. 3A; Logistic GEE; CONCENTRATION: χ^2^ = 51.733, p < 0.001; SOLUTION: χ^2^ = 4.915, p = 0.027; CONCENTRATION × SOLUTION: χ^2^ = 16.011, p = 0.003). At the lowest concentration of lead tested (0.001 mg/L) the percentage of bees exhibiting PER to the lead contaminated sucrose solution was lower than the percentage of bees responding to the uncontaminated sucrose solution (Fig. 3A; Bonferroni-corrected pairwise comparisons 0.001 mg/L: p = 0.016). Across the range of lead concentrations we tested, the percentage of bees exhibiting PER to the uncontaminated sucrose solution decreased by 38% while the percentage responding to the sucrose + lead solution decreased by only 23%, leading to a significant interaction between the effects of the metal concentration and the solution tested (Sucrose vs. Sucrose + Lead; Fig. 3A; Logistic GEE: CONCENTRATION × SOLUTION: χ^2^ = 16.011, p = 0.003). This resulted in no significant difference between the percentage of bees responding to the lead contaminated sucrose solution and the uncontaminated sucrose solution at the higher concentrations of lead tested (Bonferroni-corrected pairwise comparisons, 0.01 mg/L: p = 1.000, 0.1 mg/L: p = 1.000, 1 mg/L: p = 1.000, 10 mg/L: p = 1.000). During the proboscis stimulation assay, the concentration of lead in the contaminated sucrose solution also had a significant effect on the percentage of bees consuming the test solutions (Fig. 3B; Logistic GEE; CONCENTRATION: χ^2^ = 10.169, p = 0.038; SOLUTION: χ^2^ = 32.415, p < 0.001). For the lower concentrations of lead, the percentage of bees that consumed the sucrose + lead solution was significantly lower than the percentage of bees that consumed the uncontaminated sucrose solution (Bonferroni-corrected pairwise comparisons, 0.001 mg/L: p < 0.001, 0.01 mg/L: p = 0.019, 0.1 mg/L: p < 0.001, 1 mg/L: p < 0.001). However, the difference between the consumption of the lead contaminated sucrose and the uncontaminated sucrose decreased as the concentration of lead in the contaminated sucrose increased. At the highest concentration of lead (10 mg/L) there was no significant difference between the percentage of bees consuming the lead contaminated sucrose and those consuming the uncontaminated sucrose (Bonferroni-corrected pairwise comparisons, 10 mg/L: p = 1.000).

### The effect of ingestion of metal contaminated sucrose on the sucrose response threshold

There was no significant effect of cadmium chloride pretreatment on the bees’ ability to discriminate between sucrose and water or in their overall responsiveness to the sucrose solutions over the series of trials (Fig. 1C; MultiLog GEE; CONCENTRATION: χ^2^ = 7.367, p = 0.195; SUCROSE%: χ^2^ = 89.704; p < 0.001). All bees showed an approximately equal increase in responsiveness to increasing concentrations of sucrose, regardless of the concentration of cadmium used for the pretreatment (CONCENTRATION).

For bees pretreated with copper chloride, all treatment groups exhibited an increased percentage of bees exhibiting PER over increasing sucrose concentrations and an increasing discrimination between sucrose trials and water trials during the assay, as expected (Fig. 2C; MultiLog GEE; CONCENTRATION: χ^2^ = 5.405, p = 0.368; SUCROSE%: χ^2^ = 66.939; p < 0.001). There was, however, no effect of copper pretreatment on the percentage of bees responding to each concentration of sucrose tested (CONCENTRATION).

Ingestion of lead contaminated sucrose resulted in a small yet significant interaction between sucrose sensitivity and lead chloride treatment (Fig. 3C; MultiLog GEE; CONCENTRATION: χ^2^ = 8.240, p = 0.143; SUCROSE%: χ^2^ = 110.685; p < 0.001; CONCENTRATION × SUCROSE%: χ^2^ = 43.731, p = 0.012). All treatment groups showed the expected increasing percentage of bees responding to the increasing sucrose concentrations and increasing ability to discriminate between the sucrose and water trials as the sucrose concentration of the test solutions increased (SUCROSE%). The bees pretreated with 0.01 mg/L and 1 mg/L lead exhibited a higher responsiveness and discrimination to the 1% sucrose test trials than the control indicating a slightly higher sensitivity to sucrose and a better ability to discriminate sucrose trials from water trials for this sucrose concentration (Fig. 3C; MultiLog GEE contrasts; 0.01 mg/L at 1% sucrose: p = 0.041, 1 mg/L at 1% sucrose: p = 0.032).

## Discussion

We show that exposure to sublethal levels of three different heavy metals elicited three very different response profiles in pollen forager honey bees. These differences may reflect a varying ability to detect the substances, including variation in the sensory mechanisms involved. The differences may also be indicative of the bees’ perception of the nutritional value, if the metal is a trace nutrient, or harmful potential of the metal. Alternatively, these differences could be due to an alteration in the perceived value of the food source. Thus, each of these response profiles have differing implications for the level of threat these metals have to honey bee health and survival.

Forager honey bees did not exhibit any significant rejection of cadmium contaminated sucrose solutions at the concentrations we tested. For the highest concentrations of cadmium we tested, there was a decrease in the percentage of bees consuming the contaminated sucrose following proboscis stimulation, though it did not reach significance. This may be indicative of a higher threshold for detection via the proboscis than the concentrations we tested. Or, the bees did not perceive these low concentrations of cadmium as aversive. Electrophysiology studies of antennal and proboscis responses to cadmium stimulation are needed to determine if the lack of response is due to inability to detect the substance at these concentrations or due to the absence of a perception that the substance is aversive.

Ingestion of cadmium-contaminated sucrose also did not alter the sucrose response threshold of the animals. Di *et al*.^29^ showed that cadmium ingestion decreases the amount of sucrose consumed by adult bees 24 and 48 h following exposure but only at concentrations 5 to 10 times higher than the highest dose we used^29^. These high doses of cadmium likely induced malaise^29,31^. So, the doses of cadmium we tested were not high enough to induce malaise.

The bees showed a significant dose-dependent rejection of copper contaminated sucrose via stimulation of antennal receptors. However, the mechanisms that sense copper appear to be absent, nonfunctional, or less sensitive on the proboscis since the bees readily consumed copper contaminated sucrose following stimulation of receptors on the proboscis. To our knowledge, there are no studies identifying the mechanisms mediating the differential response to copper following antennal and proboscis stimulation. Potential hypotheses include the possibility that copper ions may alter the responsiveness of sucrose receptors found on the antennae through competitive or noncompetitive inhibition or there may be antennal receptors that are able to detect the presence of copper independently of sucrose. These mechanisms would likely be absent on the proboscis or less sensitive than those on the proboscis. An examination of the electrophysiological responses of the receptors on the antennae and on the proboscis following stimulation with copper is needed to begin clarifying the mechanisms leading to the differential behavioral responses elicited by stimulation of these sensory structures with copper.

The ingestion of copper-contaminated sucrose also did not induce any change in the bees’ sucrose response thresholds, indicating that the concentrations of copper we tested did not induce malaise within the time frame of our experiment. However, when pretreated with doses of copper at least three times higher than the highest dose than we used, forager honey bees show a decreased consumption of sucrose 24 h following ingestion of the metal^29^. Therefore, the lower doses of copper we used were insufficient to induce malaise within the time frame of our experiment. There is a possibility that malaise could have developed over a longer timeframe (i.e. 48 to 72 hours) since Di, *et al*.^29^ showed that concentrations only 1.5 times higher than the highest dose we tested did cause a decrease in sucrose consumption 48 h after ingestion of the metal.

In a natural setting, sources with these concentrations of copper contamination may not adversely affect the foraging bee. However, the range of copper concentrations (<0.06–495 mg/kg) documented in soils of agricultural regions in the United States extend beyond the concentrations observed to substantially bioaccumulate in plant tissues^7,13^. The concentrations found in plant tissues were similar to or above the doses we tested and the concentrations used by Di, *et al*.^29^ that both reduce feeding and survival in adult honey bees^29^. Therefore, it is possible that honey bees may be exposed to levels of copper contaminants sufficient to induce rejection of the contaminated resource if alternate resources are available. The mechanism mediating this selectiveness may be similar to honey bees’ propensity to reject a lower value sucrose solution when the higher value food source is available^32,33^. If no alternate resources are available, the foraging bees are more likely to gather the unpalatable resource^32,34^. Though the bees may not experience an alteration in foraging behavior, such as sucrose response thresholds, chronic exposure to copper may result in gradual accumulation within the animal and in the nest, resulting in a delayed toxic effect on foraging worker bees and the whole colony.

When presented with lead contaminated sucrose, the bees exhibited a pattern of responses that indicates there may be an interaction between the detection or perception of lead and the perception of sucrose upon stimulation of the antennae or proboscis and following ingestion of the metal. The percentage of bees exhibiting PER to antennal stimulation with lead contaminated sucrose remained fairly constant. But, the percentage of bees responding to the sucrose only trials decreased over the trials, as the antennae were stimulated with higher concentrations of lead during the sucrose + metal trials. Exposure of antennal sucrose receptors to lead may have altered the function of these sensory receptors in later trials, making the bees less able to detect the sucrose content of the solutions.

With proboscis stimulation, the initial responses to low concentrations of lead in the contaminated sucrose were significantly lower than the uncontaminated sucrose trials. However, increasing the concentration of lead in the contaminated sucrose resulted in an increase in the percentage of bees consuming the contaminated sucrose. Previous studies show that sucrose contaminated with very high concentrations (≥400 mg/L) of lead is rejected by honey bees^29^. Therefore, only a narrow range of lead concentrations appears to be palatable to honey bees. So, food sources with moderate concentrations of lead may be perceived as palatable, while lower and higher concentrations have the opposite effect of decreasing the palatability of the resource. This would result in a preference for the moderately contaminated food source over uncontaminated resources^32,33^. Since the concentrations of lead that elicited an increased preference for contaminated sucrose solutions over uncontaminated sucrose are similar to those found in plants grown in contaminated soil, it is possible that honey bees in the natural environment would preferentially forage on contaminated sources as they would be perceived as of higher value than equivalent uncontaminated sources^7,13,32,33^.

Ingestion of low or moderate doses of lead caused a subsequent increase in the sucrose sensitivity upon antennal stimulation. This is similar to what has been shown with aluminum pretreatment in free flying choice experiments in honey bees^18^. The increase in sucrose sensitivity with exposure to moderate levels of lead contamination coupled with the increased palatability of moderately contaminated sucrose solutions upon proboscis stimulation indicate that lead is likely altering the perceived value of the proffered sucrose solutions. These results also demonstrate that the effect of lead on sensory perception occurs not only during the feeding bout during which lead exposure occurs but during future feeding bouts as well.

The complex array of responses to lead contamination is likely due to some type of interference with sensory transduction or an alteration of the perception of the sucrose within the proffered solutions. In other organisms, lead has been reported to inhibit calcium signaling, which is a vital component to sensory transduction and neurotransmission^35,36^. Lead has also been documented to interfere with acetylcholine, gamma-amino butyric acid, and dopamine release, all of which are involved in sensory processing and reward valuation in the honey bee^36^. Stimulation of the antennae or proboscis exposes the sensory cells to dissolved lead ions. The repeated stimulation of the antennae may have allowed the lead ions to interact with the sensory receptor proteins or intracellular targets within sensory neurons during the initial trials, which could have altered the bees’ responses to subsequent stimulations with both sucrose + lead solutions and uncontaminated sucrose. The reversed effect of lead stimulation on bees following antennal stimulation compared to proboscis stimulation may indicate some differences in sensory detection mechanisms in these two anatomical regions. When ingested, lead may be taken up by cells in the central nervous system and be altering neural signaling within the neuromodulatory circuits involved with reward valuation^36^. Studies investigating the activity of antennal and proboscis sensory receptors upon stimulation with lead or upon stimulation with sucrose following lead ingestion would help determine the mechanisms mediating the observed response patterns in each anatomical region.

The sublethal accumulation of heavy metals in the nectar and pollen of flowering plants growing near sources of contamination can have a significant effect on pollinator health and survival. The risk a metal poses to the pollinator population can be linked to how readily the pollinator species detects and rejects the substance as aversive. Those metals that honey bees are able to detected as harmful prior to ingestion are more likely to be avoided if the bees have an alternative food source that is not contaminated^34^. Honey bees in areas contaminated with copper may be able to avoid the contaminated food sources through the avoidance response we demonstrated in this study if they also have access to uncontaminated resources. Other metals have also been shown to elicit an avoidance of the contaminated food by pollinator species. For example, studies investigating the effect of nickel and aluminum contamination on pollinator visits to contaminated flowers showed that higher metal content reduced the rate of visits by generalist pollinators, indicating an avoidance response to the contaminated food^9,16,18^.

Metals and metalloids – like cadmium and selenium – that are not detected pre-ingestion at sublethal yet toxic concentrations may be readily consumed and pose a significant threat to the health and survival of the colony^16,26^. The bees’ lack of rejection of cadmium-contaminated food is especially interesting since Di *et al*.^29^ showed that cadmium is highly toxic to the honey bee, even at the concentrations we tested. In foragers, concentrations similar to those we used significantly increased adult mortality^29^. Selenium, however, has been shown to cause a reduced state of feeding motivation and learning performance, likely from long-term post-ingestional malaise. Through conditioned taste aversion, the bees may learn to associate the malaise with sensory cues from that food source and avoid the contaminated food in the future^22,37^.

Metals – like lead – that potentially alter the sensory detection or perception of sucrose and other important food sources can have a wide array of consequences to the honey bee. The resulting complex pattern of the alteration in sensory detection and perception of food sources caused by lead exposure makes it difficult to determine the likelihood of foraging honey bees rejecting a contaminated food source during a foraging excursion. It is possible that foraging honey bees could either not differentiate between lead-contaminated and uncontaminated food or even prefer moderately contaminated resources over uncontaminated resources.

Not only do these metals have the potential to alter the foragers’ feeding and resource gathering behaviors, but they also may have broader effects on neural function if they affect cellular mechanisms central to neural signaling throughout the brain. For example, exposure to toxic levels of manganese and aluminum impaired navigation in honey bees and reduced the number of effective foraging trips they were able to make before dying^17,18^. To determine if these alterations in neural function are due to direct impairment of neural signaling or due to peripheral damage altering responsiveness to sensory cues, the post-ingestional targets of these metals must be identified.

For all of the metals we tested, larvae are much more sensitive to the metals than are adult honey bees^29,30^. Larvae exhibit significantly increased mortality at concentrations of the metals similar to or lower than the moderate or lowest concentrations we tested^29,30^. From our study, it is apparent that foragers may not discriminate between uncontaminated nectar or pollen and those contaminated with low concentrations of cadmium, copper, or lead. They might even prefer the resources moderately contaminated with lead to the uncontaminated resources. This could potentially have significant negative repercussions on the health and survival of the colony. This may be especially true as the metals accumulate within the nest over time, leading to toxic effects on the larvae and eventually adult bees.

Though adult honey bees are able to reject food contaminated with some toxic heavy metals, the toxic levels of metals and metalloids in the environment still poses a significant risk to pollinators. We have shown that worker bees consume contaminated food if the metal concentration is sufficiently low. This will increase the likelihood of the worker bees gathering those contaminated resources and would allow the metal concentration to build within the hive. Over time this could result in reductions in brood survival and impair worker health and survival. Not only is colony survival significantly impacted, individual health and normal behavior are also altered by even sublethal metal exposure. There is limited information on the concentrations of heavy metal contaminants actually available to pollinators in the natural or agricultural environment and how those concentrations are likely to build in their bodies and nests over time, so this is an important area of future research.

The high probability that contaminated areas contain significant levels of multiple metals and other toxins is also problematic, since it is very likely that these substances have joint effects, such as potentiation or antagonism, on pollinator health. Previous studies have documented the presence of multiple toxins, including heavy metals, in wax, propolis, honey, and nectar, but the effects of this nest contamination is largely unexplored^38^. Investigating the behavioral and physiological effects of sublethal exposure to these environmental contaminants individually and in mixtures is of great value.

## Methods

### Animals

Worker honey bees from colonies with open-mated New World Carniolan queens were used for all experiments^39^. Queens were purchased from commercial bee breeders in northern California. For the experiments, we collected only pollen foragers at the colony entrance as they returned from foraging flights. The use of only pollen foragers reduced the between subject variability in sucrose responsiveness, since pollen foragers generally have a high sucrose response threshold. All animals were briefly anesthetized in an ice-water bath and restrained in custom harnesses, which allowed unrestricted movement of the antennae and proboscis. Upon recovery from the anesthetization, the animals were fed to satiation with 1 M sucrose (Sigma-Aldrich, St. Louis, MO) and housed in a humidified plastic tub for approximately 24 h.

### Heavy metal contaminants

The heavy metals used in these experiments were cadmium (II) chloride, copper (II) chloride, and lead (II) chloride, which are major contaminants of soil and water surrounding urbanized and industrialized locations, in agricultural regions, and near mining and hydraulic fracturing sites^2,3,5,27,40^. The metals were found to accumulate in floral tissues of plants grown in soil contaminated with these metals^7^. For cadmium (II) chloride (99.99%; Fisher Scientific, Hampton, NH) and lead (II) chloride (99%; Acros Organics, Morris, NJ), the metal concentrations used were 0.001 mg/L, 0.01 mg/L, 0.1 mg/L, 1 mg/L, and 10 mg/L. For copper (II) chloride (99%; Fisher Scientific, Hampton, NH), the metal concentrations used were 0.002 mg/L, 0.02 mg/L, 0.2 mg/L, 2 mg/L, and 20 mg/L. Each metal was diluted to the desired concentrations in a 1 M sucrose solution. These concentrations are comparable to or less than the concentrations of these metals found in contaminated environments and measured in the floral parts of plants grown in contaminated soils^7,13^. Additionally, Di, *et al*.^29^ showed these concentrations to be sublethal to adult honey bees in survival assays^29^.

### Antennal response assay

We tested the bees’ responsiveness to antennal stimulation with heavy metal contaminated sucrose solutions. Approximately 50 min prior to beginning the assay, the bees (cadmium: n = 89, copper: n = 89, lead: n = 86) were fed 30 μl 1 M sucrose and were placed in the humidified box for 20 min. Then, the bees were fed to satiation with water and placed in the humidified box for an additional 30 min. This reduced the likelihood of the bees responding to the sucrose solutions in order to obtain the water rather than responding to the sucrose concentration alone. During the assay the bees’ antennae were briefly stimulated with the following series of stimuli: deionized water, 1 M sucrose, deionized water, 1 M sucrose + metal. Stimulation with deionized water served as to control for sensitization, the potential of residual sucrose on the antennal surface eliciting a response regardless of the solution offered, and habituation to the sucrose-containing stimuli. This series was repeated 5 times for each bee, and the concentration of metal in the contaminated sucrose solution was increased with each repetition. The presence or absence of the proboscis extension reflex in response to antennal stimulation was recorded for each trial. At no point during the assay were the bees allowed to feed.

### Proboscis response assay

We tested the bees’ responsiveness to proboscis stimulation with metal contaminated sucrose solutions. Approximately 30 min prior to beginning the assay, the bees (cadmium: n = 21/treatment group, copper: n = 23/treatment group, lead: n = 28/treatment group) were fed 30 μl 1 M sucrose. During the assay, the bee’s antennae were stimulated with 0.6 μl 1 M sucrose to elicit PER. If the bee extended its proboscis, it was fed 0.6 μl of one of the following series of stimuli: 1 M sucrose, deionized water, 1 M sucrose + metal. The small volume fed during each trial ensured that the bees would not become satiated during the assay. If the bee consumed the entire droplet of the test solution offered, its response for the trial was recorded as a “1”. If it did not consume the droplet its response for the trial was recorded as a “0”. Independent treatment groups were used for each concentration of metal in the contaminated sucrose stimuli.

### Sucrose response threshold assay

We examined the effect of ingesting metal contaminated food on the bees’ sucrose response threshold. Approximately 2 h prior to beginning the assay, 6 groups of bees (cadmium: n = 28/treatment group, copper: n = 17–21/treatment group, lead: n = 27–30/treatment group) were fed 20 μl 1 M sucrose or 1 M sucrose + metal for all metal concentrations listed above. Immediately prior to beginning the assay, the bees were fed to satiation with deionized water. During the assay, the bees’ antennae were briefly stimulated with increasing concentrations of sucrose (0.1%, 0.3%, 1%, 3%, 10%, 30%). Prior to each of the sucrose stimulations the bees’ antennae were briefly stimulated with deionized water, to serve as a control for sensitization. The presence or absence of PER was recorded for each water and each sucrose trial. At no time during the assay were the bees allowed to feed on the solutions used for stimulation.

### Statistical analysis

All statistical analyses were completed in IBM SPSS version 23. The results of the antennal response assay and the proboscis response assay were analyzed using a binary logistic regression analysis adjusted for repeated measures: Logistic generalized estimating equations (Logistic GEE). This analysis evaluates the differences in the probability of a PER response to stimulation with sucrose and to stimulation with metal-contaminated sucrose over each of the metal concentrations tested. The percentage of bees responding to water was not included in the analysis since the large difference between the percentage of bees responding to water trials and the percentages of bees responding to both sucrose (Sucrose and Sucrose + Metal) trials would have obscured meaningful differences between the Sucrose and Sucrose + Metal trials. When indicated by the data structure, second-order interaction terms between the test SOLUTIONS (Sucrose vs. Sucrose + Metal) and the metal CONCENTRATION were included in the analysis. If the interaction term was not significant, it was removed from the model and the main effects model was used. *Post hoc* pair-wise comparisons with a Bonferroni correction for multiple comparisons were used to determine which concentrations of metal significantly altered the probability of PER exhibition compared to sucrose only trials.

The results of the sucrose response threshold assay were converted to a series of discrimination code (DC) scores for each pair of water and sucrose trials before statistical analysis. The DC score describing a bee’s response to each pair of trials was calculated using the following equation:

$${\rm{DC}}={\rm{response}}\,{\rm{to}}\,{\rm{sucrose}}\,{\rm{stimulation}}-({\rm{response}}\,{\rm{to}}\,{\rm{water}}\,{\rm{stimulation}}/2)$$

This generated unique DC scores for individuals that responded to sucrose stimulation only (DC = 1), individuals that responded to water stimulation only (DC = −0.5), individuals that responded to both water stimulation and sucrose stimulation (DC = 0.5), and individuals that did not respond to either water stimulation or sucrose stimulation (DC = 0). Because there were four possible discrimination code scores (outcomes), the differences in the likelihood of each of these outcomes occurring were analyzed for each sucrose concentration (SUCROSE%) tested using a multinomial logistic regression analysis adjusted for repeated measures: Multinomial logistic generalized estimating equations (MultiLog GEE).

## Data Availability

The data presented in this manuscript are available upon request from the corresponding author.
